# MODY probability calculator utility in individuals' selection for genetic testing: Its accuracy and performance

**DOI:** 10.1002/edm2.332

**Published:** 2022-07-12

**Authors:** Tiago da Silva Santos, Liliana Fonseca, Sílvia Santos Monteiro, Diana Borges Duarte, Ana Martins Lopes, André Couto de Carvalho, Maria João Oliveira, Teresa Borges, Francisco Laranjeira, María Luz Couce, Maria Helena Cardoso

**Affiliations:** ^1^ Division of Endocrinology, Diabetes and Metabolism Hospital de Santo António – Centro Hospitalar e Universitário do Porto Porto Portugal; ^2^ Division of Pediatric Endocrinology Department of Pediatrics Centro Materno‐Infantil do Norte – Centro Hospitalar e Universitário do Porto Porto Portugal; ^3^ Centro de Genética Médica Jacinto de Magalhães (CHUPorto) Porto Portugal; ^4^ University Clinical Hospital of Santiago de Compostela, IDIS CIBERER MetabERN Santiago de Compostela Spain

**Keywords:** genetic testing, MODY, probability

## Abstract

**Introduction:**

MODY probability calculator (MPC) represents an easy‐to‐use tool developed by Exeter University to help clinicians prioritize which individuals should be oriented to genetic testing. We aimed to assess the utility of MPC in a Portuguese cohort with early‐onset monogenic diabetes.

**Methods:**

This single‐centre retrospective study enrolled 132 participants submitted to genetic testing between 2015 and 2020. Automatic sequencing and, in case of initial negative results, generation sequencing were performed. MODY probability was calculated using the probability calculator available online. Positive and negative predictive values (PPV and NPV, respectively), accuracy, sensitivity and specificity of the calculator were determined for this cohort.

**Results:**

Seventy‐three individuals were included according to inclusion criteria: 20 glucokinase (*GCK‐MODY*); 16 hepatocyte nuclear factor 1A (*HNF1A‐MODY*); 2 hepatocyte nuclear factor 4A (*HNF4A‐MODY*) and 35 DM individuals with no monogenic mutations found. The median probability score of MODY was significantly higher in monogenic diabetes‐positive subgroup (75.5% vs. 24.2%, *p* < .001). The discriminative accuracy of the calculator, as expressed by area under the curve, was 75% (95% CI: 64%–85%). In our cohort, the best cut‐off value for the MODY calculator was found to be 36%, with a PPV of 74.4%, NPV of 73.5% and corresponding sensitivity and specificity of 76.2% and 71.4%, respectively.

**Conclusions:**

In a highly pre‐selected group of probands qualified for genetic testing, the Exeter MODY probability calculator provided a useful tool in individuals' selection for genetic testing, with good discrimination ability under an optimal probability cut‐off of 36%. Further geographical and population adjustments are warranted for general use.

## Graphical abstract

This real‐world setting study validates the utility of the Exeter MODY Probability Calculator among a population with different geographical features, with good discriminative ability under a similar probability cut‐off (36%). Among our population, its 35 years at diagnosis age cut‐off constitutes a major weakness, decreasing its sensitivity. Our data suggest the need for further geographical and population adjustments to improve its performance for general use.

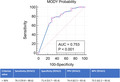

## 1 INTRODUCTION

Monogenic forms of diabetes that develop with an autosomal‐dominant inheritance are classically aggregated in the maturity‐onset diabetes of the young (MODY) categories. MODY is responsible for approximately 1%–2% of all cases of diabetes diagnosed in Europe.^1^, ^2^, ^3^ According to a nationwide population‐based study from Norway, its prevalence may reach 6.5% among childhood diabetes with negative pancreatic islet autoantibodies.^4^ Its true prevalence is thought to be largely underestimated, with around 80% of individuals with monogenic diabetes misdiagnosed as type 1 or type 2 diabetes, probably due to some overlapping phenotypic characteristics between these diabetes subtypes.^5^, ^6^ Currently known MODY subtypes are caused by dominantly acting heterozygous mutations in 11 genes that are crucial for the development or function of pancreatic‐*β*‐cells, namel*y HNF4A*, *GCK*, *HNF1A, PDX1, HNF1B*, *NEUROD1*, *KLF11*, *CEL*, *PAX4*, *INS*, *BLK*, *ABCC8*, *KCNJ11 and APPL1*.^7^ Mutations in *GCK*, *HNF1A* and *HNF4A* account for approximately 94% of cases.^5^

Genetic diagnosis is pivotal to the diabetes management of these people with diabetes given that it can help to decide the most appropriate treatment. Individuals with *HNF1A* and *HNF4A* mutations are usually sulphonylurea responsive, whereas *GCK*‐MODY rarely requires pharmacological treatment. In addition, genetic testing allows prognostic stratification of vascular complications and potential extra‐pancreatic features and family counselling and treatment during pregnancy.^8^

The traditional criteria of MODY (people with diabetes diagnosis <25 years, non‐insulin treated and an affected parent) are based on the absolute age of diagnosis cut‐offs and have shown a significant lack of sensitivity identifying less than 50% of monogenic diabetes.^5^ On the other hand, routine genetic testing without any previous individuals' selection is both inadequate and expensive.^9^ Individuals' selection for genetic testing that is based on pretest probability determined by clinical and demographic data is preferable. To address this problem, Shields and associates from the University of Exeter Medical School, Royal Devon and Exeter NHS Foundation Trust (Exeter, UK) developed in 2012 a MODY probability calculator (MPC), which consists of a validated mathematic model that generates a probability of identifying a relevant mutation in genes *HNF1A*, *HNF4A* or *GCK*. It is based only on clinical features (such as age at diagnosis, BMI, HbA1c, family diabetes history and insulin/non‐insulin hypoglycaemic agents use) and has shown good discrimination between monogenic and type 1 (T1DM) or type 2 DM (T2DM) in a European cohort of individuals diagnosed with less than 35 years of age.^8^, ^10^ We aimed to assess the accuracy of this MPC for MODY diagnosis in a Portuguese cohort with early‐onset non‐type 1 or type 2 diabetes.

## 2 MATERIAL AND METHODS

### 2.1 Study design and population

This retrospective observational study enrolled 132 participants referred to genetic testing at a Portuguese Tertiary Hospital due to clinical suspicion of MODY, during a period of 6 years, between 2015 and 2020.

Monogenic diabetic suspicion was based on the following criteria: (1) early‐onset diabetes (<35 years old); (2) negative pancreatic autoantibodies, including antiglutamic acid decarboxylase (GAD) antibody, anti‐islet cell antibody (ICA) and antizinc transporter protein 8 antibody (ZnT8); (3) persistently detectable C‐peptide plus low or no insulin requirement 2 years after the diagnosis; (4) and dominant inheritance (family history of diabetes in one parent and other first‐degree relatives of that affected parent). Exclusion criteria included the following: (1) criterion of type 1 diabetes; (2) individuals with clinical signs of insulin resistance (acanthosis nigricans, increased abdominal circumference and obesity) and (3) other types of diabetes such of the diseases of the exocrine pancreas, drug‐related or other primary endocrinopathies.

Clinical data of the patients, including age at diagnosis, gender, body mass index (BMI) at diagnosis, family history of diabetes, diabetes‐related complications and treatment options were obtained. Laboratory data at diagnosis such as plasma C‐peptide, glycated haemoglobin (HbA1c) and β‐cell autoantibodies were also collected.

This study was performed in line with the principles of the Declaration of Helsinki *and* was approved by the Ethics Committee from *Centro Hospitalar e Universitário do Porto*, Portugal. Due to the retrospective nature of the study and the absence of additional clinical procedures beyond those performed in the delivery of usual care, consent to participate was waived by the local Ethics Committee. Data were anonymized.

### 2.2 Genetic testing

Genomic deoxyribonucleic acid (DNA) was extracted from peripheral blood lymphocytes and used with custom‐designed primers for polymerase chain reaction amplification of the coding regions and exon‐intron boundaries of the GCK, HNF1A and HNF4A genes. Automated or Sanger sequencing analysis was undertaken for all individuals. Those with no pathogenic mutation identified by conventional sequencing underwent further targeted next‐generation sequencing using Clinical Exome Solution V2 ^®^ (Sophia Genetics SA). Enriched libraries were sequenced on the NextSeq platform (Illumina Inc.) following the manufacturer's recommendations using a multiplex system with 16 samples per run with the NextSeq 500/550 Mid Output V2 kit (Illumina Inc.). The genetic analysis strategy was performed with a virtual panel based on Human Phenotype Ontology consisting of 200 genes associated with familial hyperinsulinism, monogenic diabetes, neonatal diabetes and other disorders in which hypoglycaemic/ hyperglycaemic events are a predominant sign.^11^

To achieve a reliable clinical interpretation of the variants detected, and to predict their pathogenicity, we considered prioritization criteria according to American College of Medical Genetics and Genomics (AMCG) guidelines.^12^ We considered allele frequency using the exome aggregation consortium database (ExAC), 1000 Genomes Project database and gnomAD.^13^, ^14^, ^15^ Several pathogenicity algorithms were considered to predict disease by Mutation Taster and damaging by FATHMM (functional analysis through hidden Markov models) and DANN (deleterious annotation of genetic variants using neural networks) scores. According to Genomic Evolutionary Rate Profiling (GERP), PhyloP and phastCons, variants were analysed according to their positions in highly conserved regions through evolution. The clinical significance of variants was evaluated with ClinVar and polymorphism database (dbSNP).^12^

### 2.3 Measures

**FIGURE 1:**
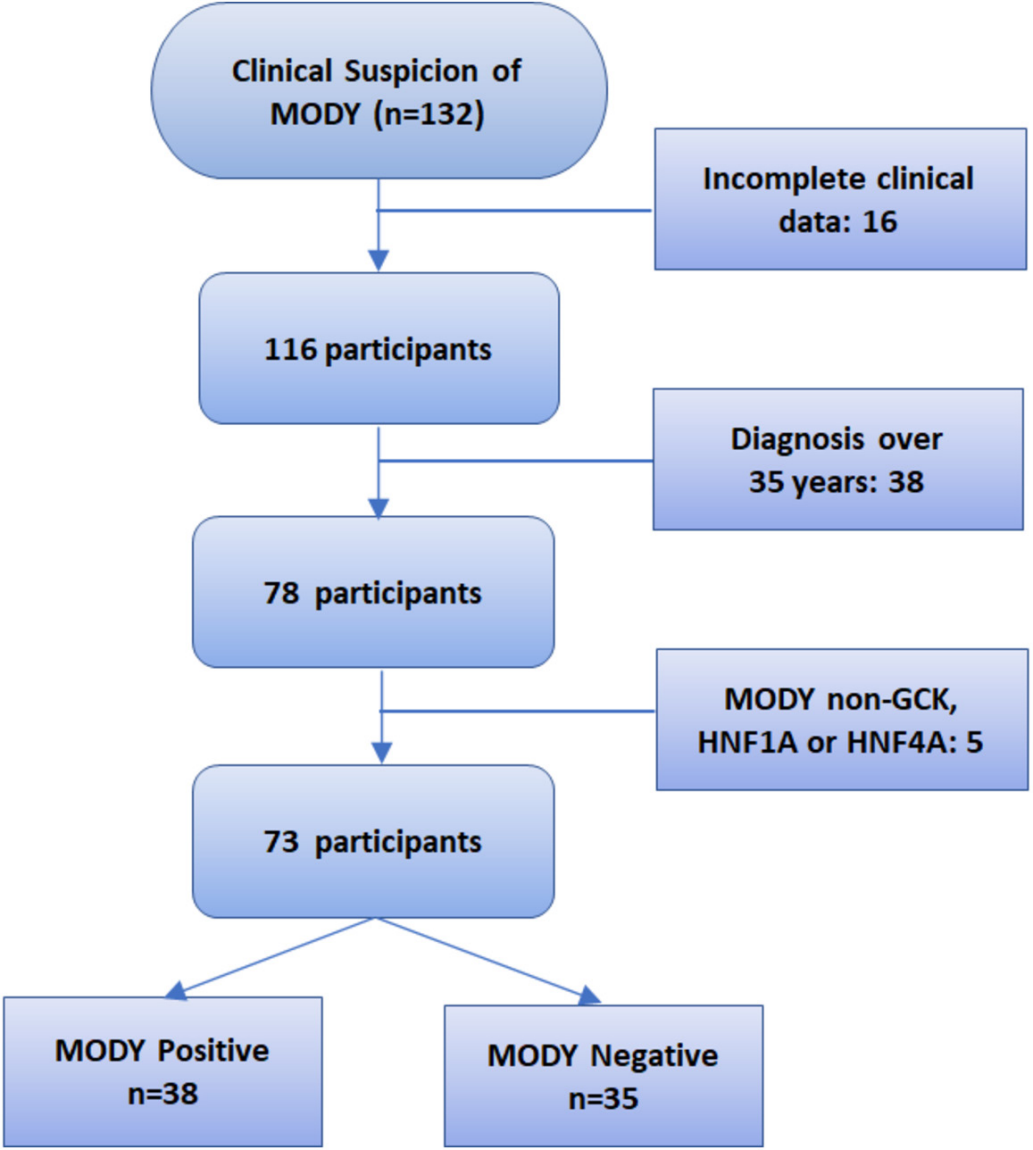
Participant's selection flowchart

Participants were stratified according to genetic findings in two subgroups: monogenic diabetes‐*positive* and monogenic diabetes‐*negative*. MODY probability was based on a formula available on the website www.diabetesgenes.org (accessed in August 2021) that uses the following parameters: patients' current age, age at diagnosis, sex, paternal family history of diabetes, BMI, HbA1c, ongoing treatment and duration of insulin treatment. Participants without complete clinical data were excluded from the analysis, and patients with diabetes diagnosis over 35 years old gave that this tool has not been validated after this age. Only individuals with mutations in *HNF4A*, *GCK* or *HNF1A* gene were included given that this calculator is only validated on these mutations. Participants' selection is summarized in Figure 1.

### 2.4 Statistical analysis

Continuous and categorical variables are presented as mean ± standard deviation (SD) or medians with interquartile ranges (IQR) and numbers with proportions, respectively. For continuous quantitative variables, distribution normality was tested through histogram observation and Kolmogorov‐Smirnov test analysis. The Student's *t*‐test and the Mann‐Whitney *U* test were used to compare continuous variables with normal and non‐normal distribution between groups, respectively. Pearson's chi‐square test was used to compare categorical data. Receiver operating characteristic (ROC) curves were plotted to determine the best cut‐off in terms of sensitivity and specificity. The discriminative accuracy of the test (calculated as the area under the curve [AUC] on the ROC curve), the positive predictive value (PPV), negative predictive value (NPV), sensitivity and specificity of the test were also calculated. All statistical tests were 2‐tailed and performed on the IBM SPSS^®^ computer statistics program, version 25. A *p*‐value of <.05 was considered statistically significant.

## 3 RESULTS

From a total of 132 participants, 73 were evaluated based on the inclusion criteria (Figure 1). Their baseline characteristics are listed in Table 1. The median age at diabetes diagnosis was 24 years (IQR:14–29), with a median diabetes duration of 18 years (7–27). Forty‐six patients were women (63%), mostly with normal weight at diagnosis (67%) and 90% of them with a known family history of diabetes. Median HbA1c was 48 mmol/mol (6.5%), median fasting C‐peptide was 1.55 nmol/L and 41% presented at least one diabetes‐related complication. Twenty‐eight patients (38%) were under insulin therapy and 27 (37%) were exclusively treated with non‐insulin hypoglycaemic agents.

**TABLE 1:** Comparison of clinical characteristics between participants with and without positive genetic testing for MODY

Participants characteristics	Total *N* = 73	MODY positive *n* = 38	MODY negative *n* = 35	*p*‐Value
Age at diagnosis (years)	24 (14–29)	17 (7–29)	27 (20–30)	.009*
Diabetes duration (years)	18 (7–27)	12 (4–23)	24 (16–33)	.002*
Women (yes), *n* (%)	46 (63)	24(63)	22 (63)	.58
BMI at diagnosis, *n* (%)				
<18 kg/m^2^	5 (7)	5 (13)	0	NA
18–24.9 kg/m^2^	49 (67)	31 (82)	18 (51)	.009*
25–29.9 kg/m^2^	11 (15)	2 (5)	9 (26)	.008*
≥30 kg/m^2^	8 (11)	0	8 (23)	NA
Family history of diabetes (yes), (*n*/%)	66 (90)	35 (92)	31 (89)	.70
HbA1c (mmol/mol)	48 (41–62)	44 (40–49)	57 (44–68)	<.001*
Fasting C‐peptide (nmol/L)	1.55 (1.04–2.10)	1.50 (1.10–1.95)	1.80 (0.99–2.30)	.52
Diabetes‐related complications (yes), *n* (%)				
Microvascular	30 (41)	6 (38)	24 (69)	<.001*
Macrovascular	11 (15)	3(19)	3 (9)	<.001*
Treatment (yes), *n* (%)				
Insulin	28 (38)	6 (16)	22(63)	<.001*
NIHA	27 (37)	20 (53)	30 (86)	.003*
Diet	18 (25)	15 (39)	0	NA
MPC (%)	58.0 (15.0–75.5)	75.5 (45.5–75.5)	24.2 (6.4–62.4)	<.001*

Note

Continuous variables are presented as mean ± SD or median (interquartile range).

Abbreviations: BMI, body mass index; HbA1c, haemoglobin A1c; MPC, MODY probability calculator; NA, not applicable; NIHA, non‐insulin hypoglycaemic agents; PPV, positive predictive value.

Thirty‐eight participants (52%) were found to harbour either pathogenic/likely pathogenic variants in *GCK* (*n* = 20), *HNF1A* (*n* = 16) and *HNF4A* (*n* = 2) genes (MODY‐positive group). Full description of the genetic variants found is available at Table S1. A total of 35 (48%) were put forward for genetic testing but were not found to have any *GCK*, *HNF1A* or *HNF4A* pathogenic mutations (MODY‐negative group). Within this subgroup, 8 of them (23%) scored over 75.5% on the calculator. MODY‐positive individuals were younger at diabetes diagnosis (17 vs. 27 years, *p* = .009) with a lower diabetes duration (12 vs. 24 years, *p* = .002) and lower median HbA1c (44 vs. 57 mmol/mol, *p* < .001). MODY‐negative participants were more frequently either overweight (26% vs. 5%, *p* = .008) or obese (only patients in this group presented a BMI ≥30 kg/m^2^) and had a higher rate of diabetes‐related complications (69% vs. 38%, *p* < .001). The former was also more frequently treated both under insulin (63% vs. 16%, *p* < .001) or other non‐insulin hypoglycaemic agents (86% vs. 53%, *p* = .003) (Table 1).

**FIGURE 2:**
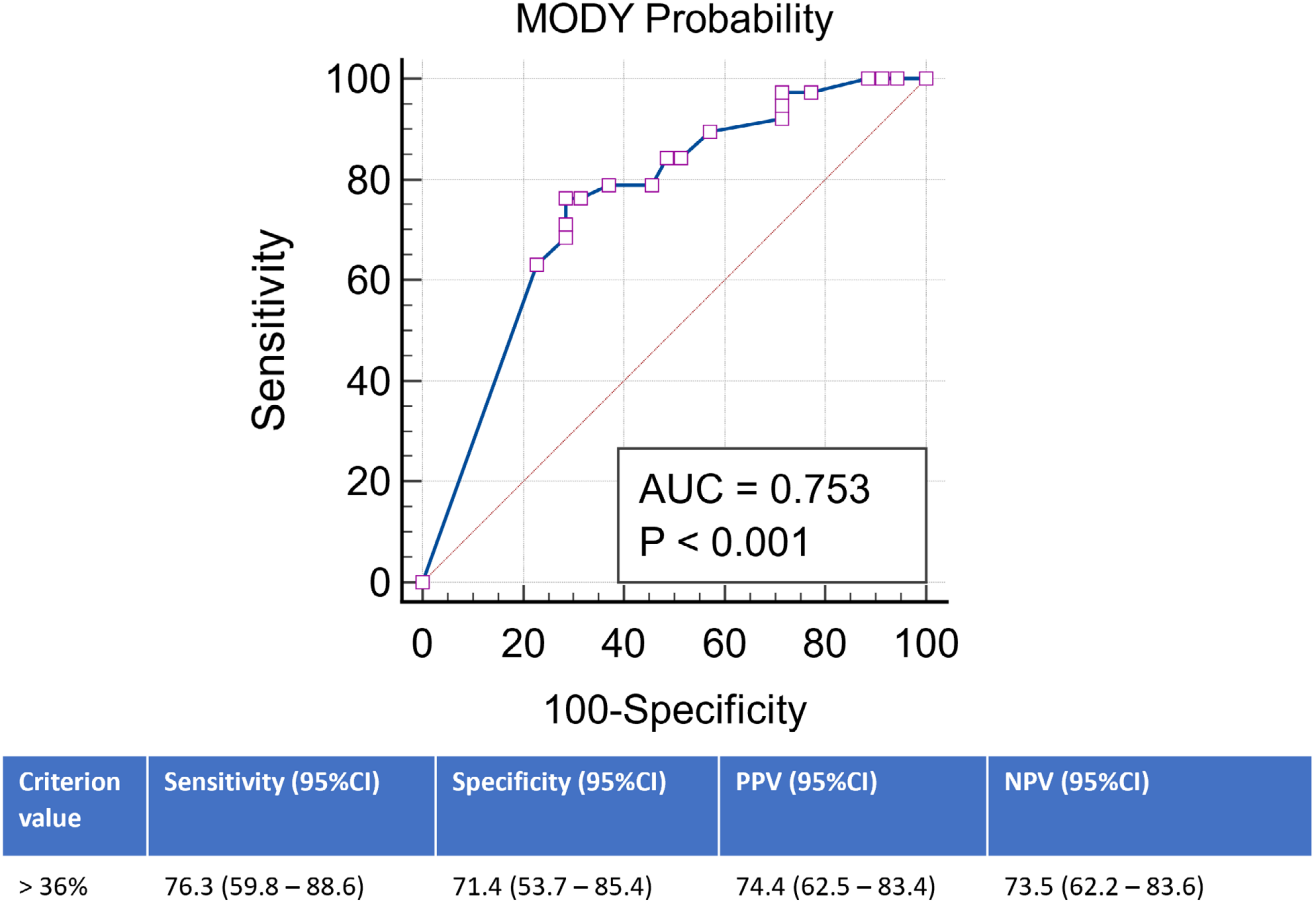
Receiver operating characteristic curve analysis of the MODY probability calculator for prediction of positive genetic testing for MODY. 95% CI, 95% confidence interval; AUC, area under the curve; NPV, negative predictive value; PPV, positive predictive value

Based on the results from MPC, our participants presented a median probability score of MODY of 58.0% (IQR: 15.0%–75.5%). Post‐test probability was significantly higher in the MODY‐*positive* vs. MODY‐*negative* individuals (75.5% vs. 24.2%, *p* < .001) (Table 1). The discriminative accuracy of MPC between *positive* and *negative* individuals for MODY was 75% (95% CI: 64%–85%). The ROC analysis best cut‐off value for the association with positive genetic testing was set at a probability over 36%, with a PPV of 74.4%, NPV of 73.5% and corresponding sensitivity and specificity of 76.2% and 71.4%, respectively (Figure 2). At this probability rate cut‐off, 26% of the cases would have been missed (false‐negative rate). The currently recommended pick‐up rate for genetic testing (MODY probability > 25%) performed poorer in our population, with a discriminative accuracy of 67%, a lower PPV (65.2%) and a higher percentage of missing cases (30%) (Table 2).^6^ If genetic testing had been limited to the traditional clinical criteria for MODY, fewer individuals would have required testing (*n* = 25), leading to a higher PPV (80%), but a higher proportion of MODY cases, which would have been missed (38%) (Table 2).

**TABLE 2:** PPV and NPV values for the MODY probability calculator (using probabilities >36% and >25% as pick‐up rate value for genetic testing) and traditional MODY criteria (age at diagnosis younger than 25 years, non‐insulin treated and parent affected with diabetes)

	PPV (%)	NPV (%)	Potential MODY cases missed (%)
MPC cut‐off point (probability > 36%)	74.4	73.5	26
Shields MPC cut‐off point (probability > 25%)	65.2	70.3	30
Traditional MODY criteria	80.0	62.5	38

Note

Data presented as percentage (%).

Abbreviations: MPC, MODY probability calculator; NPV, negative predictive value; PPV, positive predictive value.

**FIGURE 3:**
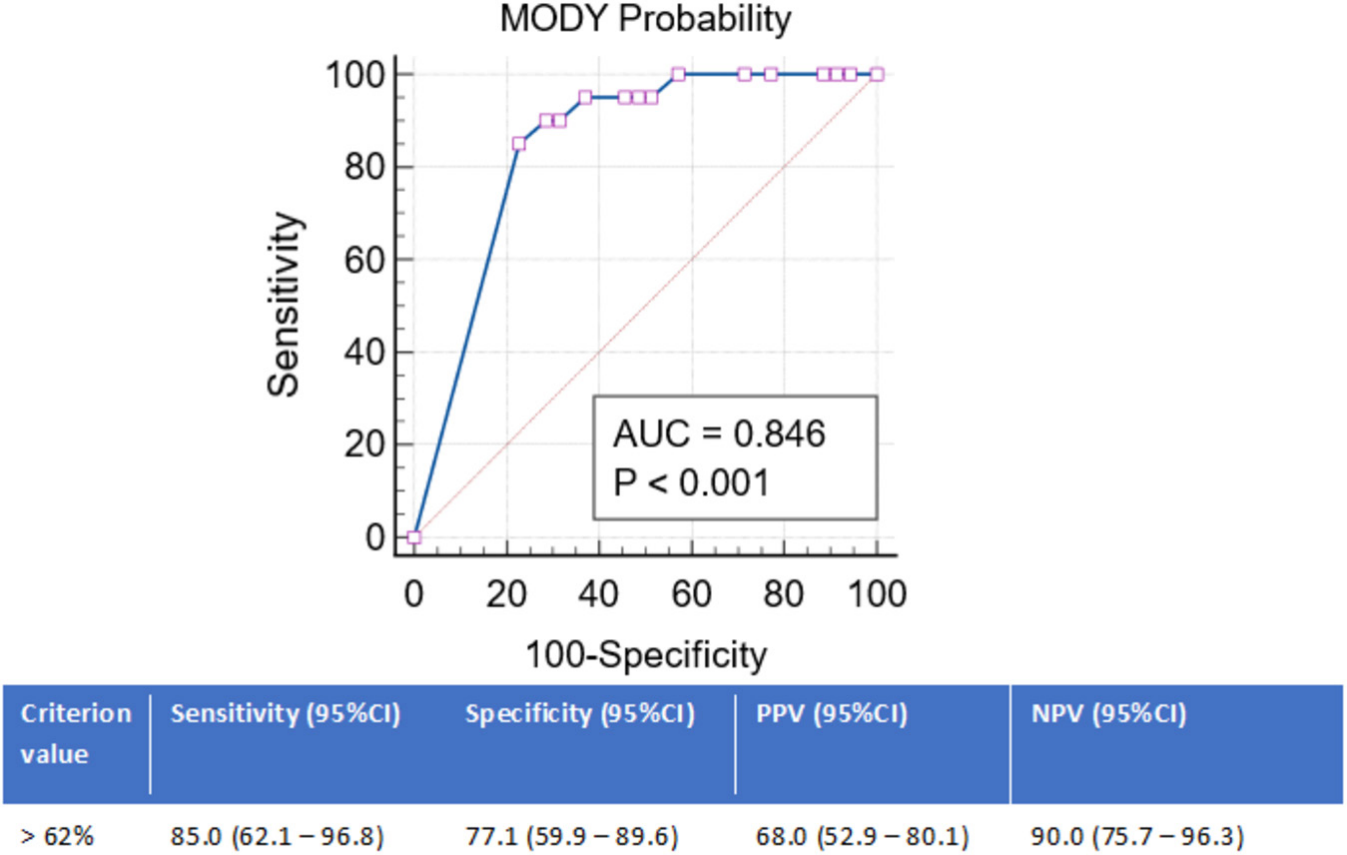
Receiver operating characteristic curve analysis of the MODY probability calculator for prediction of positive genetic testing for *GCK*‐MODY. 95% CI, 95% confidence interval; AUC, area under the curve; NPV, negative predictive value; PPV, positive predictive value

**FIGURE 4:**
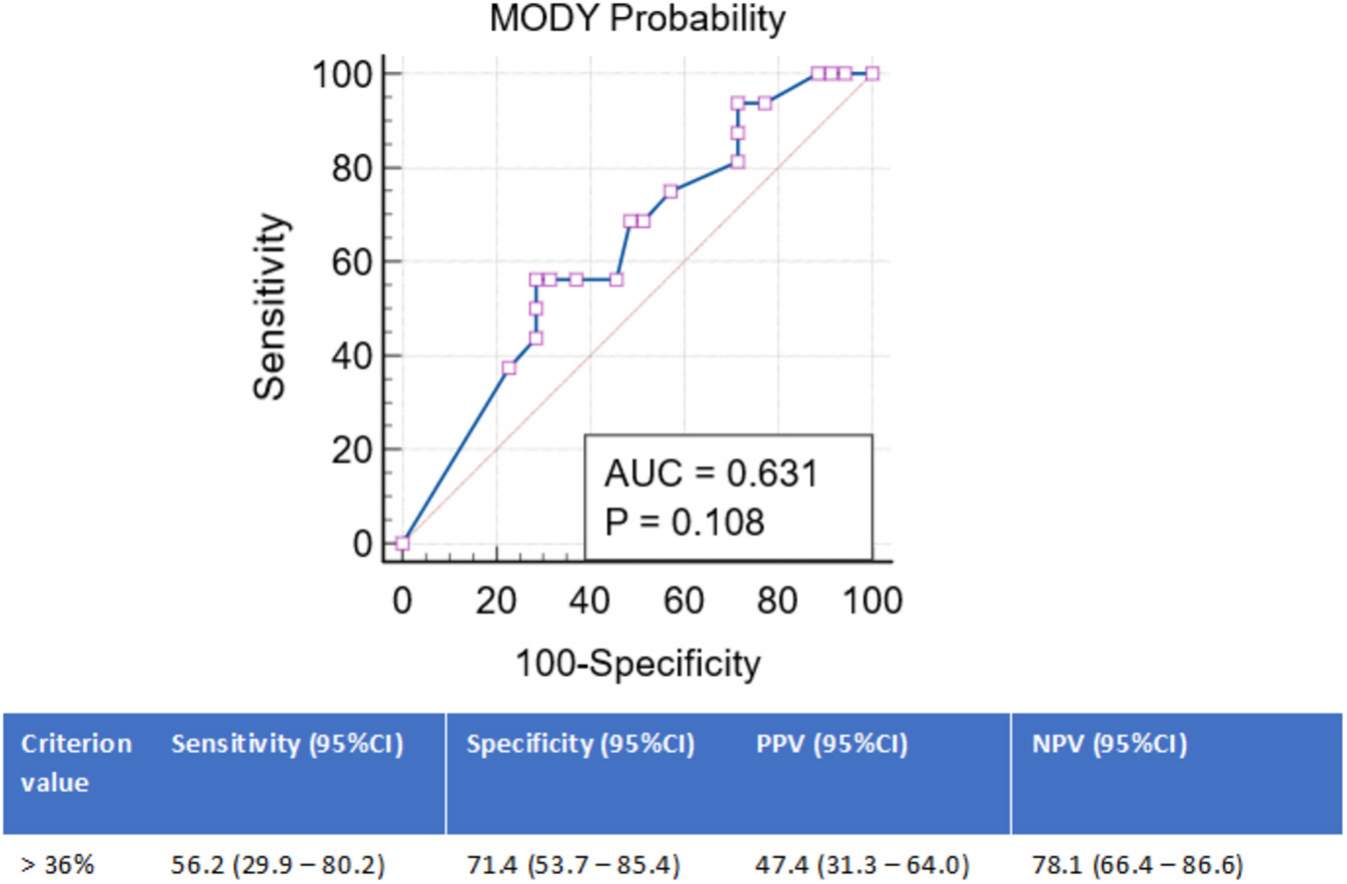
Receiver operating characteristic curve analysis of the MODY probability calculator for prediction of positive genetic testing for *HNF1A*‐MODY. 95% CI, 95% confidence interval; AUC, area under the curve; NPV, negative predictive value; PPV, positive predictive value

In a sub‐analysis, when MPC was applied to distinguish between *GCK*‐MODY (*n* = 20) and *negative* individuals for MODY, and its discriminative accuracy was 85% (95% CI: 72%–93%). The ROC analysis best cut‐off value for the association with positive genetic testing for *GCK*‐MODY was set at a probability over 62%, with a PPV of 68.0%, NPV of 90.0% and corresponding sensitivity and specificity of 85.0% and 77.1%, respectively (Figure 3). In contrast, the discriminative accuracy of the calculator between *HNF1A*‐MODY *(n = 16)* and *negative* individuals for MODY was 63% (95% CI 49%–76%) and the best cut‐off value for the association with positive genetic testing for *HNF1A*‐MODY was set at a probability over 36%, with a PPV of 47.0%, NPV of 78.% and corresponding sensitivity and specificity of 56.2% and 71.4%, respectively (Figure 4).

Thirty‐eight participants were excluded from general analysis due to diabetes diagnosis age above 35 years old. Within this subgroup, ten individuals (26%) tested positive for MODY (*HNF1A*: 7 patients, *GCK*: 3 patients).

## 4 DISCUSSION

With this unicentric cohort study, we intended to evaluate the accuracy of MPC for the screening of monogenic diabetes subtypes *GCK*, *HNF1A* and *HNF4A* MODY. Our data show that using MPC, a probability cut‐off of 36% provides the best discriminatory value for detecting individuals with true monogenic diabetes at a relatively good sensitivity (76%) and specificity (71%) (Figure 2). Particularly within *GCK*‐MODY individuals, a higher probability cut‐off (62%) may be beneficial to further increase its discriminative ability (Figure 3). We estimate that approximately 3 out of 4 individuals referred for genetic testing with MPC post‐test probability above 36% will have MODY (PPV). This MPC prediction model by Shields and associates was created based on a larger cohort of white North Europeans with a recommended cut‐off point of 25% as a reasonable value to undergo genetic testing. Among those early insulin‐treated individuals, a probability above 10% may even represent a suitable value to endorse further testing for either C‐peptide or islet cell autoantibodies.^9^, ^10^ Our study proposes a higher post‐test probability cut‐off performs better within our population. Other authors have already suggested higher cut‐off values for MODY detection.^16^ Ang et al.^17^ calculated a pick‐up rate of 62% to efficiently discriminate between MODY diabetes‐positive and diabetes‐negative participants among South‐Asian individuals. Tarantino et al.^18^ suggested that a MPC probability rate above 75% could be a cost‐effective strategy for individuals' selection to screening for non‐*GCK* MODY mutations among a Brazilian cohort. However, other studies among white European individuals have already questioned MPC's utility in clinical practice. Hohendorff et al.^19^ report that, within a highly pre‐selected group of proband from Polish families that qualified for genetic testing based on clinical features, MPC failed to improve individuals' selection for genetic testing. McCarthy et al. reinforce its limitations and high false‐negative rate within an Irish cohort, which may result from excluding both individuals aged over 35 years at diagnosis and the ones with a strong family history of diabetes but without an affected parent.^20^ Specific geographical adjustments may be necessary to further calibrate this probability model and optimize its performance, even within White‐Caucasian populations.

Within our cohort, MPC sensitivity and negative predictive value (76% and 74%, respectively) were somewhat lower than expected for a screening test, although better than traditional clinical criteria (Table 2).^2^ Several arguments may help explain these results. First, later age at diagnosis and insulin treatment initiation may partly underpower MODY probability rate within some MODY cases, further decreasing its sensitivity. Second, on the other hand, in almost 50% of participants (*n* = 35) no genetic defect was identified in either *GCK*, *HNF1A*, *HNF4A* or even among rarer MODY subtypes that were analysed by next‐generation sequencing methods. These individuals were significantly older, with a higher median HbA1c, higher need for treatment under insulin and a higher rate of diabetes‐related complications. Third, there were some individuals (*n* = 8) with MPC probability over 75% in whom no monogenic cause was identified, which significantly lowered MPC's specificity within our population. We might argue that mutation‐negative genetic tests can be explained by the occurrence of phenocopies, given that these suspected MODY cases may represent other types of diabetes that can also occur in young individuals and coexist within an affected family, thus mimicking the phenotype of MODY.^21^ Also, syndromic monogenic diabetes should be considered. Monogenic diabetes syndromes are frequently only tested when this is supported by its specific syndromic clinical features. However, recent studies have shown that syndromic monogenic diabetes genes (particularly m.3243A > G, *HNF1B and WFS1*) are more common than previously thought and usually lack their typical syndromic clinical features, presenting overlapping diabetes phenotypes with non‐syndromic monogenic diabetes. Given those partial presentations, routine testing for syndromic monogenic diabetes genes in individuals with suspected MODY must be considered.^22^, ^23^ On the other hand, monogenic diabetes‐negative participants were significantly older at diagnosis, decreasing their likelihood of a genetic cause. Lastly, sometimes these mutations may be either located in the promoter or deep intronic regions or result from large deletions of the genes that may not be detected by conventional sequencing.^24^

In particular, MPC performed better within *GCK*‐MODY individuals than within *HNF1A*‐MODY (Figures 3, 4). We might argue that this may result both from the high frequency (41%) of *GCK*‐MODY probands included in the MODY clinical prediction model development and the relatively ‘homogeneous’ clinical presentation that characterizes this MODY‐subtype, in contrast with *HNF1A*‐MODY clinical heterogeneity.^10^, ^11^, ^12^, ^13^, ^14^, ^15^, ^16^, ^17^, ^18^, ^19^, ^20^, ^21^, ^22^, ^23^, ^24^, ^25^

Our work proposes that a higher post‐test probability score (>36%) using MPC does yield a high mutation detection rate in our population. On the other hand, applying strict criteria such as ours is likely to miss a proportion of affected individuals, although significantly less than when using clinical features alone.^26^ Performance and cost‐effectiveness depend on the cut‐off values used, with lower cut‐off values resulting in higher sensitivity and costs per extra detected mutation. Its optimal threshold value may depend on both specific healthcare settings and the nature of the mutation detected. For instance, we might accept a high cost per detected mutation when a positive result may lead to a more tailored treatment approach, such as *HNF1A*‐MODY, which can be efficiently managed with sulphonylureas instead of insulin. On the other hand, a higher cut‐off value may be beneficial within *GCK*‐MODY screening, especially given its benign evolution, with no need for any treatment.^9^, ^27^ Moreover, as the costs of genetic testing are decreasing, and with the continuing drive to increase awareness on monogenic diabetes, it is realistic to expect a rise in testing for MODY and acknowledge that an accurate diagnosis of monogenic diabetes may currently reveal high cost‐effectiveness return.

### 4.1 Limitations of the MODY Probability Calculator

Some participants were excluded from our analysis given that MPC has only been validated in individuals diagnosed below 35 years of age (Figure 1). We have found that over one‐quarter of this subset of participants were positive for monogenic diabetes, mainly positive for *HNF1A* mutation. Within this specific mutation, it is already known that approximately 60% of *HNF1A* individuals developed diabetes until 25 years old, 80% until 35 years and 95% until 55 years.^28^ Thus, we might argue that these individuals either remain within the 20% diagnosed above 35 years of age or had a later diagnosis, probably due to insufficient access to the healthcare facilities. Applying a higher age cut‐off may be beneficial to increase this *HNF1A* screening test's sensitivity. Moreover, other studies already suggested that a cut‐off age at 45 years would more efficiently select participants for genetic testing in this clinical setting.^29^ Within these individuals, additional biomarkers, such as plasma C‐peptide and pancreatic autoantibodies, should be incorporated to enhance their selection.^6^, ^7^, ^8^, ^9^, ^10^, ^11^, ^12^, ^13^, ^14^, ^15^, ^16^, ^17^, ^18^, ^19^, ^20^, ^21^, ^22^, ^23^, ^24^, ^25^, ^26^, ^27^, ^28^, ^29^, ^30^

Five participants presented other monogenic diabetes mutations (*HNF1B*: *n* = 2; *PDX1*: *n* = 2; *APPL1*: *n* = 1) (Figure 1) but were excluded from the analysis. One limitation of current MPC is that it is only validated for the three most common subtypes of MODY (*HNF4A*, *GCK* and *HNF1A*) when there are at least 1 genes known to cause autosomal‐dominant monogenic diabetes. Larger studies are needed to adapt and enhance MPC's ability to pick up rarer subtypes of MODY and thereby validate its utility for all monogenic suspected individuals.^8^

### 4.2 Strengths and limitations of the study

A strong point of our work is that we present one of the first studies to evaluate MPC performance in the real‐world setting, specifically within a large cohort of Portuguese subjects diagnosed with diabetes before 35 years of age, which were evaluated by genetic testing.^31^, ^32^, ^33^, ^34^, ^35^, ^36^, ^37^, ^38^, ^39^, ^40^, ^41^, ^42^ In a highly pre‐selected cohort based on strict clinical criteria, our results showed that MPC may indeed improve participants' selection for genetic testing. Therefore, it constitutes a valuable tool to help the busy clinical when evaluating a new patient with diabetes.

This study has some limitations. First, its retrospective design should be acknowledged, with potential selection bias inflicted. Second, most of the participants evaluated were from northern Portugal where ethnic White‐Caucasian is predominant; therefore, our results should not be generalized to non‐Caucasian populations. Third, we cannot exclude the possibility that some individuals categorized as type 1 or type 2 diabetes (and therefore not evaluated) did have monogenic diabetes, as genetic testing was not carried out on them all, increasing type II error in our analysis.

## 5 CONCLUSIONS

In conclusion, the Exeter MODY probability calculator shows good discrimination between monogenic and the more common type 1 and type 2 diabetes in a highly pre‐selected group of Portuguese individuals diagnosed under the age of 35. It provides a useful tool for selecting patients for genetic testing, but its 35 years at diagnosis age cut‐off constitutes a major weakness, decreasing its sensitivity. Local cut‐off points determination and further geographical and population adjustments are warranted for general use.

## CONFLICT OF INTEREST

The authors declare that they have no conflicts of interest.

## AUTHOR CONTRIBUTIONS

**Tiago Santos:** Conceptualization (equal); Formal analysis (equal); Investigation (equal); Methodology (equal); Writing – original draft (lead); Writing – review & editing (equal). **Liliana Fonseca:** Conceptualization (equal); Data curation (equal); Formal analysis (equal); Methodology (equal); Writing – review & editing (equal). **Sílvia Santos Monteiro:** Data curation (equal); Formal analysis (equal); Writing – review & editing (equal). **Diana Borges Duarte:** Data curation (equal); Formal analysis (equal); Writing – review & editing (equal). **Ana Martins Lopes:** Data curation (equal); Formal analysis (equal); Writing – review & editing (equal). **André Couto de Carvalho:** Conceptualization (equal); Methodology (equal); Supervision (equal); Writing – review & editing (equal). **Maria João Oliveira:** Writing – review & editing (equal). **Teresa Borges:** Supervision (equal); Writing – review & editing (equal). **Francisco Laranjeira:** Writing – review & editing (equal). **María Luz Couce:** Writing – review & editing (equal). **Maria Helena Cardoso:** Project administration (equal); Supervision (equal); Writing – review & editing (equal).

## Supporting information

Table S1

## Data Availability

The data that support the findings of this study are available from the corresponding author, upon reasonable request.
