# Supplementary material for: MODY probability calculator utility in individuals' selection for genetic testing: Its accuracy and performance
Source: Endocrinol Diabetes Metab. 2022 Jul 12;5(5):e00332. doi: 10.1002/edm2.332 (PMC9471596; doi:10.1002/edm2.332)
Supplement: Supplementary file 1 — Table S1 [file EDM2-5-e00332-s001.docx]

**Suplementary Table S1.** Pathogenic/likely pathogenic variants detected in MODY-associated genes

| Gene | Exon | Patient | | Variant | Consequence | Pathogenicity | References |
| --- | --- | --- | --- | --- | --- | --- | --- |
| *HNF4A* | 8 | 1-2 | p.Gly284Pro fs*21  c.850_860delinsCCT | | Frameshift | P | Novel |
| *GCK* | 2 | 3 | p.Glu40Lys  c.118G>A | | Missense | P | [31] |
| *GCK* | 5 | 4 | p.Arg191Trp  c.571C>T | | Missense | P | [32] |
| *GCK* | 5 | 5-8 | c.579+1_579+33del33 | | Deletion | P | [33] |
| *GCK* | 6 | 9-10 | p.Thr206Pro  c.616A>C | | Missense | P | [34] |
| *GCK* | 7 | 11 | p.Leu288Pro  c.863T>C | | Missense | LP | Novel |
| *GCK* | 7 | 12 | p.Gly318Arg  c.952G>A | | Missense | P | [31] |
| *GCK* | 9 | 13-14 | p.Ser383Leu  c.1148C>T | | Missense | LP | [35] |
| *GCK* | 9 | 15 | p.Ala387Val  c.1160C>T | | Missense | LP | [31] |
| *GCK* | 10 | 16-22 | p.Phe423Tyr  c.1268T>A | | Missense | LP | [36] |
|  |  |  |  | |  |  |  |
| *HNF1A* | 1 | 23-24 | p.Gly31Asp  c.92G>A | | Missense | P | [37] |
| *HNF1A* | 2 | 25-26 | p.Arg159Gln  c.476G>A | | Missense | P | [38] |
| *HNF1A* | 3 | 27 | p.Tyr218Cys  c.653A>G | | Missense | P | [39] |
| *HNF1A*  *HNF1A* | 4 | 28-29 | p.Gly292fs  c.872dupC | | Frameshift | P | [40] |
| *HNF1A* | 4 | 30-31 | p.Pro291fs  c.872delC | | Frameshift | P | [41] |
| *HNF1A* | 6 | 32-33 | p.Leu383fs*  c.1146_1156del | | Frameshift | LP | Novel |
| *HNF1A* | 6 | 34-35 | p.Por379Thr  c.1135C>A | | Missense | P | [42] |

Pathogenic (P) and likely pathogenic (LP variants were defined according to ACMG criteria
